# Perceived stress and resilience levels during the COVID-19 pandemic among critical care nurses in Saudi Arabia: a correlational cross-sectional study

**DOI:** 10.7717/peerj.13164

**Published:** 2022-05-06

**Authors:** Wafa Almegewly, Albatoul Alhejji, Lama Alotaibi, Malak Almalki, Maha Alanezi, Amal Almotiri, Fai Alotaibi, Seham Alharbi, Atheer Albarakah

**Affiliations:** 1 Department of Community Health Nursing, College of Nursing, Princess Nourah bint Abdulrahman University, Riyadh, Saudi Arabia; 2 College of Nursing, Princess Nourah bint Abdulrahman University, Riyadh, Saudi Arabia

**Keywords:** Resilience, Stress, Critical care nurses, Covid-19, Saudi Arabia

## Abstract

**Background:**

The continuous spreading of the respiratory coronavirus disease, COVID-19, has been a threat to global health, especially among those fighting directly against it. Nurses who work in critical care have reported very high levels of stress during these extreme circumstances. It is very important to measure the level of stress and resilience among these nurses in order to diminish further psychological distress. This study aims to assess the levels of perceived stress and resilience among critical care nurses.

**Methodology:**

In this correlational cross-sectional study, critical care nurses (*n* = 139) were recruited by gatekeepers in a governmental university hospital in Riyadh City between 12 March and 8 April 2021 to complete an online questionnaire. The measurement tools used in this study were the Connor-Davidson Resilience Scale 10 (CD-RISC-10) and the Perceived Stress Scale of COVID-19 (PSS-10 items). Data were analyzed using a descriptive and inferential analysis to calculate frequencies to determine the distribution of stress and resilience, and multiple regression was applied to assess the relationship between them.

**Results:**

One hundred and thirty-nine critical care nurse (64%) responded. The perceived levels of stress reported were: no stress (8%; *n* = 12), mild stress (14%; *n* = 21), moderate stress (38%; *n* = 55), high stress (22%; *n* = 32), and severe stress (18%; *n* = 26). The levels of resilience reported were: very low (8%; *n* = 11), low (18%; *n* = 26), moderate (42%; *n* = 62), and high (32%; *n* = 47). The level of stress and resilience reported by the majority of critical care nurses was moderate; there was no significant correlation between COVID-19-related stress and resilience among the critical care nurses. Severe levels of stress were mostly reported among critical care nurses working in the NICU and high levels of stress were reported among those working in the emergency department. The nurses reported being highly confident that they were able to handle personal epidemic related problems with a mean score of 2.36. This reflects having a high level of resilience (42%; *n* = 62) and was significantly associated with years of experience as a nurse (*p* < 0.0027).

**Conclusion:**

Although COVID-19 cases had declined significantly during the study period in Saudi Arabia, the majority of nurses were still experiencing moderate to high levels of stress about the epidemic, but were, at the same time, moderately resilient. Continued monitoring of the stress levels of this high-risk group is highly essential. Conducting more research is needed to measure the effectiveness of psychosocial support interventions.

## Introduction

The whole world was dramatically changed when the World Health Organization (2020) announced the outbreak of the novel Coronavirus Disease 2019 (COVID-19), which was originally discovered in Wuhan, China (Zhu et al., 2020a) and quickly spread, becoming a worldwide pandemic (Li et al., 2020b). COVID-19 is highly contagious and has a two to 14-day incubation period. Even during the interval between suspecting and confirming a case, an infected patient might transmit the disease (Lauer et al., 2020), which makes it very challenging to slow down the disease’s spread. After 12 weeks, the pandemic spread internationally in 66 nations around the world (Arab News, 2020), and the (Saudi Ministry of Health (MOH)) declared the first case of COVID-19 in the country on March 2, 2020 (Saudi Arabia’s Ministry of Health, 2020). Since then, the COVID-19 outbreak has expanded to over 206 cities in Saudi Arabia, infecting 365,099 individuals and causing 6,329 fatalities (Saudi Arabia’s Ministry of Health, 2022). Saudi authorities banned sports activities, closed educational institutions, parks, and shopping centers, as well as domestic public transportation, and implemented partial curfews (Yezli & Khan, 2020; Alshammari, Altebainawi & Alenzi, 2020). Saudi Arabia began adopting early preventative measures and used one of the strictest strategies to curb the COVID-19 pandemic limiting both human and economic losses (Shimul et al., 2021). These measures included: blocking international borders and closing the two main mosques in Mecca and Medina to both native and foreign religious visitors, closing all mosques for Friday prayers, and closing all government offices except vital service providers (Mahmud & Al-Mohaimeed, 2020). A year after the initial outbreak of COVID-19, nurses were still staidly set on the front line facing the extreme pressure of taking care of critically ill patients. Due to their stressful workload, high level of responsibility, and complexity of care, critical care nurses experience high levels of stress compared to nurses in other clinical settings even before the starting of COVID-19 pandemic. Emergency department nurses in India have also reported a high risk of burnout due to an excessive workload and high amount of critical patient cases (Jose, Dhandapani & Cyriac, 2020; Windsor-Shellard, 2017).

Nurses tend to disregard their own interests during unforeseen natural disasters and viral outbreaks to willingly participate on the front lines and also make self-sacrificing decisions out of a moral and ethical responsibility (Maben & Bridges, 2020).

Several Saudi studies were conducted during COVID-19 among health care providers reporting high levels of stress and anxiety (Alotni & Elgazzar, 2020; Mohsin et al., 2021; AlJhani et al., 2021; Alenazi et al., 2020), specifically among those who were on the front lines (AlHanawi et al., 2020). During COVID-19, nurses were highly affected by depression in comparison to their physician colleagues (Al Mutair et al., 2021). According to a cross-sectional healthcare worker survey conducted in 17 countries including Saudi Arabia aimed at assessing factors linked with psychological distress including fear of COVID-19 and reported coping levels, physicians experienced higher psychological distress, but had a lower fear of COVID-19, while nurses reported moderate to high resilient coping levels (Rahman et al., 2021). This psychological distress is caused by many factors including: the high risk of infection dealing directly with COVID-19 patients, witnessing numerous deaths, an overwhelming workload, shortages of protective supplies, family isolation, and physical exhaustion (Nobles et al., 2020; Chew et al., 2020; Zhu et al., 2020b; Brooks et al., 2020). Several studies found that young age and female gender were predictive risk factors of depression among healthcare workers (Mohsin et al., 2021; Slama et al., 2021; Alkhamees et al. 2020). The level of depression among participants aged 31–40 years old was substantially greater than the level of depression among participants aged 50 and older (Al Mutair, Alhajji & Shamsan, 2021). Occupational status, number of family members, and years of experience in the medical sector were all linked to burnout (AlJhani et al., 2021). However, coping mechanisms were found to be more prevalent in those aged 25–34 years, females, married, and those with a bachelor’s degree (Natividad et al., 2021).

Psychological resilience refers to a person’s ability to adapt to major stressors such as psychological or physiological trauma, threats, tragedies, familial and relationship troubles, job stress, and financial concerns (American Psychological Association, 2012). Nurses need resilience as they face multiple stressors on a daily basis and have to deliver quality care to their patients under all circumstances. The rapid spread of COVID-19, the increase in the number of patients hospitalized, and intensive contact with critically ill patients all put critical care nurses at high risk for many psychological problems. This study adds to the body of literature involving the early identification and prevalence of stress among healthcare workers and their ability to adjust based on individual personality traits and psychological functions. This study is essential as it assesses both the stress and resilience levels among this high-risk population.

## Materials and Methods

This is a descriptive correlational cross-sectional quantitative study conducted among a convenience sample of nurses working in critical care units. The data was collected during the COVID-19 pandemic in Riyadh City, Saudi Arabia, including nurses from the emergency room (ER), the adult intensive care unit (ICU), the pediatric intensive care unit (PICU), the neonatal intensive care unit (NICU), the operating room (OR), the post-anesthesia care unit (PACU), and the cardiac surgery OR (CSOR). Ethical approval was granted from Princess Nourah bint Abdulrahman University to carry out the study within its facilities (Ethical Application Ref: 21-0055). The participants gave informed consent to take part in the research and were ensured the right to withdraw from the study. All the written informed consent forms were received from all participants.

A total of 217 questionnaires were sent to the email addresses of critical care nurses from the research unit at the selected hospital. A total of 139 critical care nurses completed the online questionnaire giving a response rate of 64%.

### Data collection methods

We used the Perceived Stress Scale of COVID-19 (PSS-10), which contains 10 elements (Pedrozo-Pupo, Pedrozo-Cortés & Campo-Arias, 2020), to measure the perceived stress levels of study participants. Each element has a maximum score of 4 (Always) and a minimum score of 0 (Never). Elements n. 1, 2, 3, 6, 9, and 10 are scored from 0 to 4; elements n. 4, 5, 7, and 8 are scored reversely, from 4 to 0. The total assessment scores range from 0 and 40: scores between 0 and 13 are considered to be low stress, 14–26 are considered moderate, while scores equal to 27 or greater are considered high perceived stress in relation to COVID-19.

The second tool we used in this study is the Connor-Davidson Resilience Scale 10 (CD-RISC-10) (Campbell-Sills & Stein, 2007). This scale contains 10 items and serves mainly as a measure of hardiness, with items corresponding to flexibility (1, 2 and 4), sense of self-efficacy (3, 4), ability to regulate emotion (8, 10), optimism (6 and 9) and cognitive focus/maintaining attention under stress (7). Each element is scored on a five-point scale ranging from 0 to 4, with 0 indicating that the resilience statement is not at all true and a score of 4 representing that the statement is true nearly all of the time. The total score is obtained by adding up all 10 elements and can range from 0 to 40. Higher scores indicate higher resilience, whereas lower scores indicate greater difficulty bouncing back from adversity. The lowest quartile (about 1% to 25% of the population) had a score of 0–29. The second quartile (those who scored between 26% and 50%) received scores of 30 and 32. Between 33 and 36 were scored by the third quartile (51–75% of the population). The highest quartile (76–100%) had a score of 37–40 (Campbell-Sills & Stein, 2007).

### Data analysis

A statistical analysis was performed using the IBM SPSS, version 22. (SPSS Inc., Chicago, IL, USA). Descriptive statistics, specifically percentage, was used to determine the distribution of the respondents according to their demographic profile including: age, marital status, years of work experience, and level of education. Mean scores were used to determine the level of COVID-19 related stress and outlook, and percentage was used to define the distribution of the level of stress experienced by the respondents. Percentage was also used to determine the distribution of the respondents according to resilience level. A multiple regression analysis was used to determine whether the demographic characteristics of the respondents had a linear relationship to their level of COVID-19 related stress and resilience.

## Results

### The demographic profile

The demographic profile of the study participants, presented in Table 1, was as follows: a majority (60%) were between the ages of 30 and 40, followed by ages 25–30 (27%), then 45 years and older (8%), and only 5% were aged 20–25. The majority (52%) of the respondents were single, and 41% were married, while only 5% and 2% were divorced and widowed, respectively. Nearly half (42%) of the respondents had worked in healthcare between 6 and 9 years, followed by 10–15 years (25%), 3–5 years (16%), 15+ years (11%), and very few had worked less than 2 years (4%). The level of education of the respondents was overwhelmingly (81%) a bachelor’s degree, while only 10% were diploma graduates, and 9% had a master’s degree. Not one respondent had a doctoral degree. Our demographic survey also showed that 86.4% of the respondents took care of a patient with COVID-19.

**Table 1:** Demographic profile of the respondents.

Profile	Frequency	Percentage
Age		
20–25	7	5
25–30	39	27
30–45	88	60
>45	12	8
Marital status		
Single	76	52
Divorced	2	2
Widowed	7	5
Married	60	41
Level of education		
Diploma	14	10
Bachelor	119	81
Master	13	9
Doctoral	0	0
Years of work experience		
Under one year	3	2
1–2 years	6	4
3–5 years	23	16
6–9 years	61	42
10–15 years	37	25
>15 years	16	11
Patient care experience		
With COVID-19	126	86
Without COVID-19	20	14

### Level of perceived stress during COVID-19 and its correlation to demographic profile

The correlation data between stress levels and demographics (presented in Table 2) shows that the nurses who were single (19.8%) had moderate levels of COVID-19 related stress and the majority of those with moderate levels of stress (30.1%) were those with a bachelor’s degree. The correlation between levels of COVID-19 related stress of the nurses when grouped according to their years of work experience found that those with less than 1 year of work experience had mild levels of stress while the highest number of those with 1–2 years of experience (2.7%) and those with 3–5 years of experience (5.4%) both had moderate levels of stress. Moderate stress levels were also the most commonly reported among those with 6–9, 10–15, and more than 15 years of experiences (15%, 9.5% and 4.1% respectively). Nurses with 6–9 years of work experience comprised the majority (3.4%) of those who reported no stress to very low, mild (4.1%), moderate (9.5%), and high (13%) levels of stress. The highest number (9.5%) of those who reported severe levels of stress were those with 10–15 years of work experience. There were also varying levels of stress across the clinical area of assignments. The largest number of nurses who reported severe stress worked in the NICU, and the highest number of those who reported high levels of stress worked in the ED. This shows that even nurses who have not taken care of patients with COVID-19 still experience work-related stress that varies from very low to severe levels.

**Table 2:** Level of stress and its association with participant demographical profiles.

Level of stress when grouped according to	No stress (%)	Mild (%)	Moderate (%)	High (%)	Severe (%)	Sig.
Marital status						0.7138
Single	2.7	9.5	19.8	8.9	8.9	
Divorced/Widow	0	0	4	0.6	0.6	
Married	4.7	4.7	13.6	11.6	8.2	
Level of educational						0.2299
Diploma	0.6	1.7	4.7	2.7	1.7	
Bachelor	5.4	11.6	30.1	15.7	15	
Masters	2	1.7	2.7	2	0	
PhD	0	0	0	0	0	
Work experience						0.0309
Under 1 year	0	2.0	0	0	0	
1–2	0	0.6	2.7	0.6	0	
3–5	2.0	2.0	5.4	2.7	2.0	
6–9	3.4	4.1	15	13	5.4	
10–15	0.6	3.4	9.5	2.7	9.5	
<15	1.3	2.0	4.1	2.7	0.6	
Clinical area						
OR	4.7	4.1	5.4	3.4	3.4	
ED	0	4.7	15	6.8	2.7	
NICU	1.6	1.3	6.1	4.1	4.1	
PACU	1.6	0	0	1.3	0	
ICU	1.6	2	6.8	1.3	3.4	
PICU	1.6	0.6	1.3	2	0.6	
Intervention	0	0.6	0.6	0.6	0.6	
CS1	0	1.6	2	0.6	1.3	
Patient care experience with Covid-19						0.415
No	1.7	1.7	4.1	4.1	2.7	
Yes	6.8	12.3	33.5	17.1	15	

Table 2 also shows the results of the predictive tests between the demographic characteristics of the respondents and levels of COVID-19 related stress. The data show that age has a *p* value = 0.4528 which is greater than the alpha = 0.05 which is not statistically significant and indicates strong evidence for the null hypothesis. The data also show similar findings with marital status with *p* = 0.7138; educational status with *p* = 0.2299; whether or not the respondent took care of a patient with COVID-19 with *p* = 0.415; and clinical area with a *p* = 0.1343. All respective *p* values are greater than the alpha = 0.05 indicating non statistical significance. The non-significance means that the null hypothesis is not rejected which indicates that age, marital status, education status, and whether or not the nurses took care of a patient with COVID-19 do not predict the level of COVID-19 related stress among the nurses.

Table 3 below shows the level of stress reported by the specific domains of COVID-19 related stress. The nurses reported being very stressed that something serious would happen unexpectedly with the epidemic as shown by a mean score of 3.47. They also reported being highly stressed by the epidemic itself as shown by a mean score of 3.57. The participating nurses reported being moderately stressed with the inability to control the important things in their lives due to the pandemic (mean score: 3.31), the inability to cope with what must be done to control possible infection (mean score: 2.91), that things related to the epidemic were out of their control (mean score: 3.15), and that the difficulties accumulated during the epidemic made them feel unable to overcome them (mean score: 2.96).

**Table 3:** Level of stress related to specific domains of COVID-19 related stress.

COVID-19 related stress domains	*n*	SD	Mean score	Level of stress
“I have felt affected as if something serious will happen unexpectedly with the epidemic”	146	1.15	3.47	Severe
“I have felt that I am unable to control the important things in my life due to the epidemic”	146	1.23	3.31	Moderate
“I have been nervous or stressed by the epidemic”	146	1.02	3.57	High
“I have felt unable to cope with the things I have to do to control the possible infection”	146	1.18	2.91	Moderate
“I have been upset that things related to the epidemic are out of my control”	146	1.18	3.15	Moderate
“I have felt that the difficulties accumulate in these days of the epidemic and I feel unable to overcome them”	146	1.15	2.96	Moderate

Our survey results also showed the COVID-19 related positive outlook of the nurses as illustrated in Table 4. The nurses were highly confident that they were able to handle personal epidemic related problems with a mean score of 2.36. They reported a moderately positive outlook that things were going well with the epidemic with a mean score of 2.58; a moderate positivity about feeling able to control the difficulties that appeared in their lives due to the infection with a mean score of 2.72; and a moderate positivity that they had everything under control in relation to the epidemic as shown by a mean score of 2.85.

**Table 4:** COVID-19 related positive outlook of the nurses.

COVID-19 related positive outlook	*n*	SD	Mean score	Level of positivity
“I have been confident about my ability to handle my personal epidemic related problems”	146	.90	2.36	High
“I have felt that things are going well (optimistic) with the epidemic”	146	1.12	2.58	Moderate
“I have felt that I can control the difficulties that could appear in my life due to the infection”	146	1.04	2.72	Moderate
“I have felt that I have everything under control in relation to the epidemic”	146	1.02	2.85	Moderate

### Level of resilience

The largest number of nurses (62/146 or 42%) had moderate levels of resilience with scores ranging from 21 to 30, while 47 nurses (32%) had a high level of resilience as shown by scores ranging from 31 to 40. There were 26 nurses (18%) who had low resilience levels; and 11 (8%) which had very low or poor resilience as shown in Table 5.

**Table 5:** Level of resilience of the nurses.

Mean scale	Level of resilience	Frequency	Percentage
0–10	Very low	11	8
11–20	Low	26	18
21–30	Moderate	62	42
31–40	High	47	32
		*n* = 146	100

### Correlation between COVID-19 related stress, COVID-19 related positive outlook, and resilience

Table 6 shows the correlation matrix of resilience, COVID-19 related stress and positive outlook and resilience. The *r* (144) = 0.07 < *t* = 1.976 for COVID-19 related stress and positive outlook. This *r* value indicates that the values are not significant, hence, the null hypothesis is not rejected. This means that there is negligible positive correlation between COVID-19-related stress and a positive outlook on the COVID-19 pandemic.

**Table 6:** Correlation matrix (COVID-19 related stress, COVID-19 related positive outlook, resilience).

	COVID-19 related stress				
	*r*	df	alpha	Critical value	Significance
COVID-19 related positive outlook	0.07	144	0.05	1.976	Negligible positive correlation
Resilience	−0.05				Negligible negative correlation

**Note:**

The correlation is significant at The *p*-value is <0.01; *p* < 0.05 (two-tailed); Correlation matrix (COVID-19 related stress, COVID-19 related positive outlook, resilience).

Furthermore, the table also shows that the *r* (144) = −0.05 < *t* = 1.976 for COVID-19-related stress and resilience. This also indicates that there is a negligible negative correlation between COVID-19-related stress and resilience among the nurses. The null hypothesis is therefore not rejected.

## Discussion

The results of this study showed that the majority of critical care nurses reported a moderate to high level of COVID-19-related stress, similar to previous studies (Rahman et al., 2021; Mohsin et al., 2021; Alotni & Elgazzar, 2020; Crowe et al., 2021; Said & El-Shafei, 2020; Lu et al., 2020). Our study revealed that nurses were severely stressed that “something serious will happen unexpectedly with the epidemic,” which indicates that nurses may have been afraid that they could transmit COVID-19 to a loved one, a fear potentially exacerbated by the insufficient supply of personal protective equipment (Natividad et al., 2021). Also, working at a hospital that hosts COVID-19 patients, regardless of whether or not healthcare workers dealt directly with these COVID-19 patients, was significantly associated with developing anxiety (Alenazi et al., 2020).

Interestingly, our results showed that the majority of nurses had a moderate to high level of resilience, in line with studies done in Philippines and Saudi Arabia respectively (Labrague & Santos, 2020; Rahman et al., 2021). A positive correlation was found between high levels of stress and more years of experience as a nurse. This is contrary to what was stated in several studies which highlighted a connection between fewer years of work experience and an increased risk of post-traumatic stress symptoms among health care employees (Mohsin et al., 2021; Slama et al., 2021; Li et al., 2020a). This might be due to the dependency of health intuitions on those who were more qualified to take care of patients suffering from COVID-19 (AlJhani et al., 2021). The majority of nurses who reported high levels of stress were working in emergency departments. This result seems to be consistent with other research which found that nurses who worked in respiratory, emergency, infectious disease, and ICU departments were 1.4 times more likely to experience fear, as well as twice as likely to experience anxiety and depression (Li et al., 2020c, Zhu et al., 2020a). Finally, this study revealed that there is no significant correlation between COVID-19-related stress and resilience, unlike the previous study (Setiawati et al., 2021). A possible explanation for these results could be because of the timeframe in which the data was obtained. Our data was collected after curfew ended in Saudi Arabia and COVID-19 cases had significantly declined due to affective vaccine campaigns and following WHO precautions (Shimul et al., 2021). It is possible that at this point in the pandemic, the stress levels of healthcare workers declined, and resilience levels started to increase due to the successful implementation of governmental health protocols.

### Recommendations

We propose the following recommendations based on the findings of our study: We suggest further studies on critical care nurses paired with psychological consultations. We also recommend that administrators report every 6 months on the stress levels of staff nurses and decide, based on the results, what interventions should be pursued. Finally, we suggest preparing workshops for nurses to help enhance psychological resilience.

### Limitations

One of the limitations of this study was a low response rate due to the unavailability of critical care nurses. These nurses lacked the time to fill out the questionnaire because of the overwhelming workload in their units.

### Implications for practice

– Provide a psychologist to each nursing team for early psychological assessment and intervention.
– Assign appropriate patients based on nursing ability and provide any additional training required.
– Educate nurses about the safe usage of protective equipment, waste management, and sterilization.
– Ensure nurses get enough rest and are not overloaded with work.

## Conclusions

In conclusion, this study has shown the effect of the COVID-19 pandemic on the mental well-being of critical care nurses. Our study found a moderate level of stress and resilience among the nurses involved in the study. There was also no correlation between stress and resilience. The evidence from this study suggests that the mental health status of nurses working in critical care matters, and we recommend that psycho-social support services be available to those at high risk of stress. More research is also needed to determine the factors affecting the levels of stress and resilience among critical care nurses.

## Supplemental Information

10.7717/peerj.13164/supp-1 Supplemental Information 1 Raw data.

10.7717/peerj.13164/supp-2 Supplemental Information 2 Questionnaire.

## Additional Information and Declarations

### Data Availability

The following information was supplied regarding data availability: The raw measurements are available in the Supplemental File.
