# Supplementary material for: Perceived stress and resilience levels during the COVID-19 pandemic among critical care nurses in Saudi Arabia: a correlational cross-sectional study
Source: PeerJ. 2022 May 6;10:e13164. doi: 10.7717/peerj.13164 (PMC9083530; doi:10.7717/peerj.13164)
Supplement: Supplemental Information 2 [file peerj-10-13164-s002.docx]

**Demographical information**

We are nursing students 4^th^ year from nursing collage we want to assess the level of stress among critical care nurses and its relation to resilience during covid-19 and).

As a part of our graduation research. There is no direct benefit to the participants, but the society will benefit from the findings that might arise from the results You will answer a questionnaire contains of 30 questions; it will take about 7 minutes. All your information in this study will remain confidential, no names or personal identifier will be displayed, and the result of this study may be published or used in a presentation. Your participation in this study is completely voluntary. You have the right to refuse to take part in the research or drop out at any time without penalty.

Part 1:

Demographical data:

Age :

17-20 years

21-24 years

25-28 years

29+ years

Gender :

Female

Male

Marital status :

Single

Married

Separate

Divorced

Widow

Do you have children :

Yes

No

Level of education :

High school graduated

Diploma degree

Bachelor degree

Master degree

Doctoral degree

Years of working experience:

Under a year

1-2 years

3-5 years

6-9 years

10-15 years

15+ years

Where do you live :

Home

Dormitory

With relatives/ friends

Other :

Working area:

ER

Medical ICU

Surgical ICU

Pediatric ICU

OR

Have you took care of patient with COVID-19?

Yes

No

Other

Connor-Davidson Resilience Scale (CD-RISC)

|  | 0  (Not true at all) | 1  (Rarely true) | 2  (Sometimes true) | 3  (Often true) | 4  (True nearly all the time) |
| --- | --- | --- | --- | --- | --- |
| **Emotion Regulation**  1-I am able to handle unpleasant or painful feelings like sadness , fear , and anger  2-I have a strong sense of purpose in life.  3-I feel in control of my life.  4- I believe I can achieve my goals , even if there are obstacles. |  |  |  |  |  |
| **Dealing with stress**  5-I have at least one close and secure relationship that helps me when I am stressed  6-Having to cope with stress can make me stronger  7-During times of stress / crisis, I know where to turn for help.  8-Under pressure , I stay focused and think clearly  9-I am able to adapt when changes occur |  |  |  |  |  |
| **Solving problems**  10-In dealing with life's problems, sometimes you have to act on a hunch without knowing why.  11- I prefer to take the lead in solving problems rather than letting others make all the decisions.  12-When there are no clear solutions to my problems , sometimes fate or God can help |  |  |  |  |  |

**COVID-PSS-10 items**

| 1. **I have felt affected as if something serious will happen unexpectedly with the epidemic.** 2. **I have felt that I am unable to control the important things in my life due to the epidemic.** 3. **I have been nervous or stressed by the epidemic.** 4. **I have felt unable to cope with the things I have to do to control the possible infection.** 5. **I have been upset that things related to the epidemic are out of my control.**   **6- I have felt that the difficulties accumulate in these days of the epidemic and I feel unable to overcome them** | **0**  **(never)** | **1**  **(almost never)** | **2**  **(occasionally)** | **3**  **(almost always)** | **4**  **(always)** |
| --- | --- | --- | --- | --- | --- |

| 1. **I have been confident about my ability to handle my personal epidemic related** 2. **I have felt that things are going well (optimistic) with the epidemic** 3. **I have felt that I can control the difficulties that could appear in my life due to the infection.** 4. **I have felt that I have everything under control in relation to the epidemic.** | **4**  **(never)** | **3**  **(almost never)** | **2**  **(occasionally)** | **1**  **(almost always)** | **0**  **(always)** |
| --- | --- | --- | --- | --- | --- |
